# The risk of acute events among patients with sickle cell disease in relation to early or late initiation of care at a specialist center: evidence from a retrospective cohort study

**DOI:** 10.1186/s12887-020-02270-y

**Published:** 2020-08-08

**Authors:** Angela Edna Rankine-Mullings, Twila Mae Logan, Lesley-Gaye King, Colette Andrea Cunningham-Myrie, Clive Robert Scott, Jennifer Marcelle Knight-Madden

**Affiliations:** 1 Sickle Cell Unit, Caribbean Institute for Health Research, University of the West Indies, Kingston, Jamaica; 2 Mona School of Business and Management, University of the West Indies, Kingston, Jamaica; 3 Department of Community Health & Psychiatry, University of the West Indies, Kingston, Jamaica

**Keywords:** Sickle cell disease, Red blood cell disorder, Penicillin prophylaxis, Acute chest syndrome, Acute splenic sequestration

## Abstract

**Background:**

The greatest disease burden of sickle cell disease occurs early in life. Understanding factors that reduce disease related events in this period is therefore important. Hence, we assessed the impact of early care at a specialist center on the incidence of acute events during the first five years.

**Methods:**

This was a retrospective cohort study among Jamaican children with sickle cell disease. Medical records of patients born January, 2004 to December, 2009, who were registered at the Sickle Cell Unit, a specialist care facility, were abstracted for dates of initiation to care, first occurrence and frequency of the outcomes of interest (vaso-occlusive crises, acute splenic sequestration, acute chest syndrome, and infection). Patients were classified according to whether initiation of care was before (early) or after 5 months of age (late). Using standardized t-tests, χ2 tests, and a multiple-failure survival analysis the rates of acute events between groups were compared.

**Results:**

Of the total study group (*n*= 290), homozygous sickle cell disease accounted for 97% and 95% of the early (*n*=113) and late groups (*n*=177) respectively. The mean age of presentation in the early and late group was 0.2 and 2.3 years (*p*<0.01), with a mean length of follow-up of 5.2 and 3.2 years respectively (*p*<0.01). Vaso-occlusive crisis (*n*=880) and acute chest syndrome (*n*= 571) together accounted for 91.6% of the total number of events (*n*=1584). The risk of vaso-occlusive crisis and acute chest syndrome (among patients who presented with these acute events) was significantly higher in the “late” group, by 43% (Incidence rate ratio, (IRR) = 1.43, *p*<0.001); 95% CI (1.18-1.72) and 40% (IRR=1.40. *p*=0.002), 95% CI (1.12-1.75) respectively compared to “early” group. There was no difference in risk between groups for acute splenic sequestration and infection among persons presenting with these events.

**Conclusion:**

The risk of acute events in children with sickle cell disease exposed to early care at a specialist care is significantly less. Therefore, widespread screening with rapid referral to a specialist center stands to reduce substantial morbidity in Jamaica and other regions with high prevalence of sickle cell disease.

## Background

Sickle cell disease (SCD) is associated with multiple acute events. These events include vaso-occlusive crisis (VOC), acute chest syndrome (ACS), acute splenic sequestration (ASS) and infection [1, 2]. Patients with the haemoglobin (Hb) genotypes homozygous sickle cell disease, (HbSS), sickle βeta thalasaemia^0^, (HbSβ^0^) and haemoglobin SO_Arab_, (HbSOA) are known to have more severe disease compared to those with milder genotypes such as haemoglobin SC, (Hb SC), and sickle βeta thalassemia^+^, (HbSβ^+^) [3]. Among affected populations, the greatest disease burden occurs early in life and can be reduced by interventions that aim to reduce the frequency and severity of early events in persons with sickle cell disease [4].

The implementation of interventions such as disease education and penicillin prophylaxis among children with homozygous sickle cell disease in the 1980 s and newborn screening (NBS) has led to significant improvements in mortality when past and present cohorts have been compared in Jamaica [5]. These interventions are well established at the Sickle Cell Unit (SCU) in Kingston, Jamaica [6, 7].

Parental education encourages early recognition of symptoms associated with VOC, ACS, ASS and infection. Results from the Jamaica Sickle Cell Cohort study show that training parents to detect splenomegaly reduced fatalities of ASS from 28–3% of reported ASS episodes [5].

Prevention of invasive pneumococcal disease (IPD) includes penicillin prophylaxis and pneumococcal immunization [8–12]. Pneumococcal immunization includes both a conjugated pneumococcal vaccine and a polyvalent pneumococcal vaccine. While the latter has been available very early in Jamaica the former became available at the SCU in 2010 hence this cohort of children (who were born between 2005 and 2009) did not have had access to it as infants, however they would have benefitted from a catch-up schedule when it became available. Penicillin prophylaxis is commenced from the age of four months of age [6]. In Jamaica, “specialist” care for SCD falls under the purview of the SCU throughout most of the island. Haematologists have limited involvement in the care of persons with SCD.

Survival estimates for children with SCD have improved over recent years, [13, 14] as evidenced by the fact that, mortality in children < 5 years of age with SCD diagnosed at birth and managed at a comprehensive care clinic in Jamaica is no worse than that of the general population [15]. The impact of early compared to late care on the incidence acute events in persons with sickle cell disease largely unexposed to disease modifying therapies such as hydroxyurea or chronic blood transfusion therapy, is unclear. There are very few studies that examine the incidence of acute events in patients with sickle cell disease in relation to age at treatment initiation.Thus, our study was undertaken to determine the difference in the incidence of acute events before 5.5 years of age between patients with early and late treatment initiation.

## Methods

### Study design and population

We assessed the impact of early interventions on the incidence of acute events in a retrospective cohort of Jamaican children diagnosed with sickle cell disease. The study population was restricted to patients registered at the SCU. The database was queried between January 2015 and May 2016 to identify all children with date of birth between January 2005 and December 2009. Each patient’s clinical history from birth to 5.5 years was ascertained from the SCU chart, discharge summaries, and hospital notes when discharge summaries were unavailable or inadequate. Other morbidity indicators examined were proportion of patients who were: splenectomised, admitted to hospital and ICU in particular, and who experienced episodes of VOC requiring strong opioids, ACS requiring oxygen and ASS requiring transfusion. Patient visits which occurred less than 2 weeks apart due to the same event were counted as the same event.

### Patient classification

Patients were categorized into two groups for comparison of rates of acute events and other morbidity indicators. Those whose age at first visit was before 5 months were classified as “early presenters” and those whose age at first visit was 5 months or older were classified as “late presenters”. The first visit represents the date of registration to the clinic. If the patient was sick at this time, this was also documented as the date of the first presentation of an acute event. The age of five months was chosen as the cut-point as patients are rarely symptomatic prior to this time [6]. Only patients with haemoglobin (Hb) genotypes with similar disease severity were included, homozygous sickle cell disease, (HbSS), sickle βeta thalassemia^0^ (HbSβ^0^) and haemoglobin SO_Arab_ (HbSOA).

### Acute events

The incidence of four potentially severe acute events was compared. These included: VOC, ACS, ASS and infection. VOC was defined as a painful event attributed to SCD, and required medical attention either in an emergency department, or at the SCU, whether or not a hospital admission was required. An episode of ACS was defined as a history of fever and or respiratory symptoms with supporting radiological findings; those diagnosed as ACS without a supporting chest radiograph were excluded. An episode of ASS was defined as an enlargement of the spleen with a fall in haemoglobin concentration of greater than or equal to two grams/dL from baseline. All cases of infection had to be supported by a positive culture report.

### Interventions

A standard set of interventions were offered to all patients at registration at the first visit. These were parent/caregiver disease education, splenic palpation to identify splenic enlargement, as well as pain and fever management. Genotype confirmation at nine to twelve months of age was important especially for those with HbSS pattern in order to rule out HbSβthal. Infection prevention strategies implemented, included the administration of conjugated pneumococcal vaccines for all genotypes from age six to eight weeks, and penicillin prophylaxis beginning at four months of age for severe genotypes [6].

### Statistical analysis

Standardized t-tests (for continuous variables) and χ2 tests (for categorical variables) were used to determine if there were any differences in the distribution of the early and late groups. A multi-failure survival analysis was carried out on data from all patients in both groups. The event time was defined as the period between registration (time of first visit) and the event in years and the analysis time defined as the period from age of first visit to age 5.5 years. A multi-failure survival analysis was necessary as some patients had more than one episode of each event.

Stata Version 12 was used for the statistical analyses [16].

### Ethics approval

The study protocol was reviewed and approved by the Ethics Committee of The University of the West Indies and the Ministry of Health through the four Regional Health Authorities in Jamaica.

## Results

**Fig. 1:**
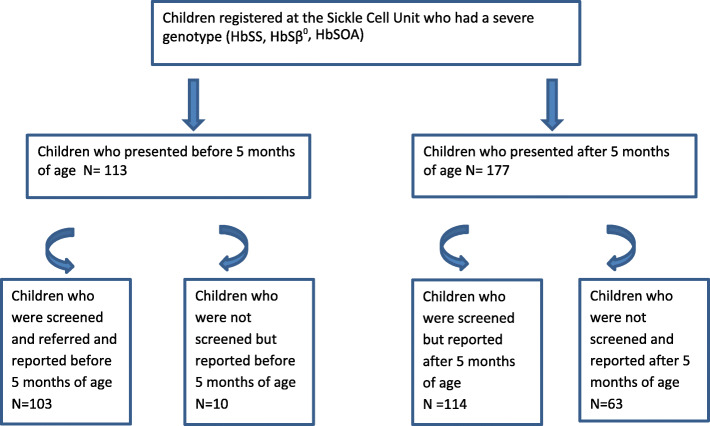
Flow diagram showing the proportion of early or late presenters diagnosed by newborn screening. N represents the number of patients

Figure 1 provides details on the 290 patients identified from records. The early group had 113 patients compared to 177 in the late group In the early group 92% (103) of patients were diagnosed by NBS compared to 64% (*n* = 114) of the late group (*p* < 0.010).

Table 1 shows further details of patients by genotype, mean age, follow up time and percentage uptake of targeted interventions in children. HbSS was the most common severe genotype. Two persons with HbSO presented late (1%). There were six (6) deaths, one in the early group and five (5) in the late group. The mean age of first visit for early presenters was 0.2 years (2.4 months) while that of late presenters was 2.3 years (27.6 months) (*p* < 0.01) with 5.2 years follow-up for the early group compared with 3.2 years for the late group (*p* < 0.010).

**Table 1:** Description of study patients by genotype, mean age, follow up time and percentage uptake of targeted interventions by early or late presentation to the Sickle Cell Unit in Jamaica (*N* = 290)

	Early Intervention (*N* = 113)	Late Intervention (*N* = 177)	
SS	110 (97%)	168(95%)	
Sβ^0^	3 (3%)	7 (4%)	
S0A	0	2 (1%)	
Mean Age at first visit, years(months)	0.2 (2.9)	2.3 (27.6)	t = 17.0; *p* < 0.010
Follow up time (years)	5.2	3.2	t= -17.0; *p* < 0.010
Proportion of patients diagnosed by newborn screening	103 (92%)	114 (64%)	χ2 = 90.6; *p* < 0.010
Proportion receiving Penicillin Prophylaxis	111 (98%)	134 (76%)	χ2 = 28.4; *p* < 0.010
Proportion receiving polyvalent pneumococcal vaccine	106 (93%)	156 (88%)	χ2 = 2.5; *p* = 0.111
Proportion with at least 2 doses PCV	35 (31%)	50 (28%)	χ2 = 0.2 ; *p* = 0.619

Ninety-eight percent (*n* = 111) of the early group received penicillin prophylaxis compared with only 76% (134) in the late group (*p* < 0.010).

However, the percentage of patients receiving pneumococcal polyvalent vaccine (*p* = 0.11) and at least two doses of conjugated pneumococcal vaccine (PCV) (*p* = 0.6190) was similar across both groups.

Table 2 shows the incidence rates of acute events and measures of morbidity in the retrospective cohort. Vaso-occlusive crisis (*n* = 880) and ACS (*n* = 571) were the most common acute events diagnosed. Vaso-occlusive crisis (*n* = 880) and ACS (*n* = 571) together accounted for 91.6% of the total number of events (*n* = 1584). There were significantly lower incidences of ACS and VOC for the early initiation of intervention group. Specifically, incidence rates for the late group for both ACS and VOC were 800/1000 person -years and 1000/1000 person-years, respectively compared to 570/ 1000 person-years and 700/ 1000 person-years for the early group (*p*-values < 0.010). Acute splenic sequestration (*n* = 112) and infection (*n* = 21) were relatively rare events. The differences in incidence rates for ASS and infection between the early and late groups of patients were not statistically different. We found no significant differences by early vs. late presenters in any of the other indicators of morbidity listed in Table 2.

**Fig. 2:**
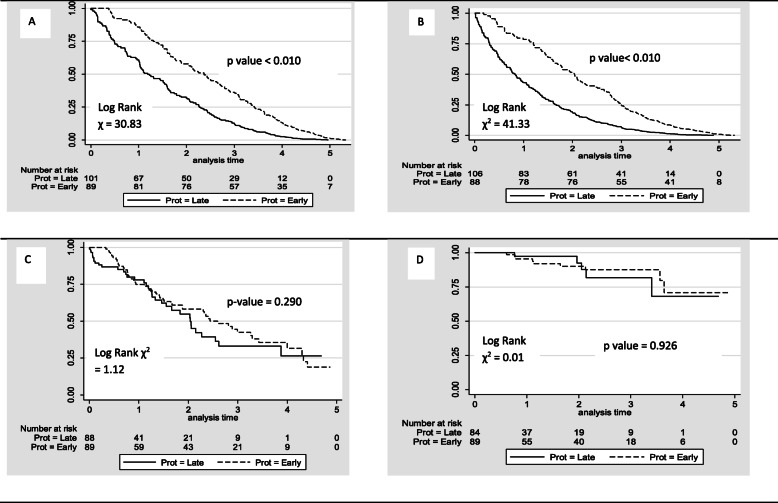
Kaplan-Meier survival curves showing incidences of acute events. Kaplan-Meier survival curves showing incidences of Acute Chest Syndrome (**a**); Vaso-occlusive crisis (**b**); Acute Splenic Sequestration (**c**) and Infection (**d**) in patients born between January 1, 2005 and December 31, 2009 who presented early or late to the Sickle Cell Unit in Jamaica and were followed from time of imitation of care at a specialist center to age 5.5years (*N* = 290)

We examined time to event for our four key outcomes with a multi-failure analysis (see Kaplan Meier survival curves, Fig. 2). The highest risk period for all events except infection was in the first 2years of life (Fig. 1). The risk of presenting with VOC and ACS, was 43% (*p* < 0.001); 95% CI (1.18–1.72), and 40% (p = 0.002), 95% CI (1.12–1.75) higher in “late” patients among patients presenting with these events. In patients presenting with ASS and infection the difference in risk between groups was not significant.

**Table 2:** Comparison of incidence rates of acute events and morbidity indicators in patients who presented before (early) or after 5 months of age (late) to the Sickle Cell Unit in Jamaica (*N* = 290)

Acute Events And morbidity indicators	Early Presenters Late Presenters Test Statistics		
Events with Incident rates (events/1000 person-year) per group			
VOC (*n* = 880)	700	1000	IRR = 1.43; *p* < 0.001 95% CI(1.18–1.72)
ACS (*n* = 571)	570	800	IRR = 1.40; *p* = 0.002 95% CI (1.12–1.75)
ASS (*n* = 112)	280	340	IRR = 1.21; *p* = 0.38795% CI (0.76–1.89)
Infection (*n* = 21)	50	50	IRR = 0.93; *p* = 0.923 95% CI (0.24–3.08)
Other indicators of morbidity (%) of patients per group (number of patients)			
Number of patients splenectomised	8 (7%)	12 (7%)	χ2 = 0.1 ; *p* = 0.950
Hospital admissions/Total visits	330 (48%)	430( 51%)	χ2 = 0.8 ; *p* = 0.364
Number Ever needing strong opioid for VOC/Total VOC episodes	48 (14%)	97 (21%)	χ2 = 5.1 ; *p* = 0.077
Number Ever requiring ICU admissions/Total visits	0	3 (0.4%)	χ2 = 3.7 ; *p* = 0.161
Number of patients needing Oxygen for ACS/Total ACS visits	59 (52%)	81( 56%)	χ2 = 0.4 ; *p* = 0.519
Transfusions for ASS/Total ASS visits	21 (36%)	15( 28%)	χ2 = 1.0 ; *p* = 0.308

*IRR* Incidence Rate Ratio

Sepsis (*n* = 09) accounted for 53% of confirmed cases of infection recorded. The frequency of meningitis (*n* = 04) and osteomyelitis (n = 04) were equal. The organisms implicated included were pneumococcus (*n* = 01) which occurred in the late group only, Salmonella (*n* = 03) and other organisms (*n* = 07). Of note there were no cases of haemophilus influenza Type B infection

## Discussion

Our aim was to determine the risk of specific acute events in patients with sickle cell disease based on early or late initiation of intervention. We found that the risk of ACS and VOC were substantially and significantly lower among children for whom care was initiated prior to age five months. However, the risk of ASS and infection did not differ significantly between groups. A comparison of morbidity in children with early and late treatment initiation was only found in one previous study by Vichinsky, carried out in Oakland, California, United States of America. [17] The findings of this study differed to ours; in the former study no differences were found in in the number of life-threatening events in patients who were identified in the NBS program compared to those diagnosed after three months. Differences in the method between studies included the fact that the time chosen as the cut point between groups was earlier than that selected in our study. Additionally, while in our study the milder genotype of haemoglobin SC was not included, this genotype was included in the group identified by newborn screening in the study being compared. Patients with Haemoglobin SC were not considered to have the same baseline risk as patients with HbSS of developing the outcomes of interest and therefore would make comparison between early and late groups difficult.

We also found that VOC and ACS accounted for the vast majority of events and occurred with high rates in our study. The incidence rate of VOC in both early and late presenters in event per person- years that was found in our study was very similar to that found in a larger study, where the incidence rates of VOC was 0.8 episode per patient-year in HBSS and 1.0 episode per patient-year in sickle beta 0-thalassemia in a group of patients ranging from newborn to 66 years of age [18]. This study found additionally, that the number of pain episodes per year was associated with early deaths in patients over 20 years of age who have sickle cell disease. This suggests that a high degree of morbidity can be averted with early initiation of care. These rates concur with VOC being the most common event experienced by children with SCD in other settings [19, 20].

Acute chest syndrome was the second most common event in both groups. The longitudinal Jamaican Cohort Study found ACS to be a major cause of death in persons with sickle cell disease in all age groups [7]. Reducing the incidence of both of these events by early interventions offered at a specialist center may therefore reduce substantial morbidity and mortality in Jamaica and other regions of the world with high prevalence of SCD even in populations without access to chronic transfusion therapy or hydroxyurea.

There was no difference in the severity of these events as evidenced by morbidity indicators such as frequency of admissions, ACS requiring oxygen, number of events that require strong opioids and the proportion of ASS requiring transfusion. This could indicate that while the incidence of events were significantly different between groups the severity were similar.

The number of episodes of ASS, though relatively less frequent was far more frequent in our study compared to a previous study in California where there was only 10 episodes over a longer study period [17]. Even though there was no significant reduction in the incidence of ASS in the early group, previous studies have shown that educating parents to palpate for the spleen daily has reduced mortality [5].

The number of confirmed cases of infections in both groups was small and similar to that seen in the study referred to previously [17]. Specifically, there were no cases of haemophilus influenza type B and the only case of Pneumococcus was not from the early group. It was possible that some patients may have received antibiotics before presentation to hospital which may have led to negative cultures being obtained.

Our study also highlights the finding that nearly two-thirds of the patients who presented late were actually screened for sickle cell disease (SCD) as newborns. Of 290 patients included in the study most were screened for SCD at birth including 64% of the “late” group. This indicates that even though children were identified as having SCD at birth via NBS and referred to SCU, they did not report for the first visit. This may have been due to challenges with contact tracing which reduced early enrolment of infants for example incorrect or indecipherable addresses and telephone numbers, or the family’s reluctance to follow-up when contacted, hence the reason for “late” entry into care at a specialist center. Despite resource constraints during the study period, the SCU attempted to trace infants who had a positive screening result. However, there is now a robust national newborn screening programme in Jamaica since 2015 of all births. Additionally the ability to trace patients has increased considerably through a national network. Newborn screening coverage in Jamaica increased to 98% of births after 2015, but it is reportedly as low as 45% in other Caribbean countries [21] and still non-existent in others.

There is need for more aggressive efforts to trace infants diagnosed with SCD and enroll them into care in a timely manner. The study demonstrates that early enrolment and subsequent early initiation of care may be an additional benefit and can only be successful if affected newborn babies are identified and parents present to and remain under the care of medical facilities with the capacity to offer lifesaving interventions. Implementation and expansion of NBS therefore needs to be accompanied by stronger linkage and retention programs for parents and their children if the demonstrated benefits of NBS [5] are to be fully realized. There is on-going work to improve linkage and retention programs for parents and their children such as training of health care workers across the island to assist in early enrolment of infants who are positive at NBS screening. Additionally, a status card will be added shortly to the health passport that is given to each infant at birth.

In spite of these challenges, survival estimates with SCD have improved over recent years [5]. In fact, mortality in children less than 5 years of age with SCD diagnosed at birth and managed at specialist care center in Jamaica is no worse than that of the general population [15]. Our retrospective cohort data provide part of the evidence for how this has been achieved, and how incremental improvements can be made and monitored. The risk of acute events in children with sickle cell disease exposed to early intervention was found to be significantly less. Therefore, widespread screening with rapid referral to a specialist center stands to reduce substantial morbidity in Jamaica and other regions with high prevalence of sickle cell disease. The authors suggest that this may be because physicians at specialist centers are able to monitor patients regularly and intervene with preventive and management options early and also because the physician can communicate with the parents/family on a regular basis and is able to encourage them to monitor the child and to seek care immediately.

### Limitations

Of note, approximately, 20% of hospital notes were not accessed for reasons which included unavailability at the time of data entry and lack of resources to retrieve further data. These omissions would more likely under count incidences of events, thus the results presented here are conservative. In some settings radiographical support was not easily accessed and microbial culture reports were not available to confirm ACS or infections. The number of ASS events and infection cases were low which may have impacted the power of the statistical analyses.

## Conclusions

The incidences of VOC and ACS were lower among children for whom care was initiated early. Our data strengthens the evidence that NBS with early intervention are essential to reduce the morbidity of sickle cell disease, even in the most resource-constrained settings.

## Data Availability

Data generated or analysed during this study are included in this manuscript and are not publicly available.

## Abbreviations

VOC: Vaso-occlusive crisis.
ASS: Acute splenic sequestration.
ACS: Acute chest syndrome.
NBS: Newborn Screening.
SCD: Sickle cell disease.
Hb: Haemoglobin.
HbSS: Homozygous sickle cell disease.
HbSβ0: Sickle βeta thalasaemia0.
HbSOA: Haemoglobin SOArab.
Hb SC: Haemoglobin SC.
HbSβ+: Sickle βeta thalassemia+.
IPD: Invasive pneumococcal disease.
SCU: Sickle Cell Unit.
PCV: Conjugated pneumococcal vaccine.
IRR: Incidence rate ratio.
CI: Confidence interval.
Prot: Protocol
